# Smoking As an Outcome Moderator In the Treatment of Alcohol Use Disorders

**DOI:** 10.1093/alcalc/agac027

**Published:** 2022-05-20

**Authors:** Jan van Amsterdam, Wim van den Brink

**Affiliations:** Department of Psychiatry, Academic Medical Center University of Amsterdam, P.O. Box 22660, 1100 DD Amsterdam, The Netherlands; Department of Psychiatry, Academic Medical Center University of Amsterdam, P.O. Box 22660, 1100 DD Amsterdam, The Netherlands

## Abstract

**Aims:**

To clarify whether smoking interferes with successful treatment of alcohol use disorder (AUD).

**Methods:**

The current systematic review investigates the potential moderating effect of smoking on behavioural and pharmacological treatment of AUD. In addition, this review summarizes the results of randomized controlled trials investigating the effect of smoking cessation treatments in subjects with AUD on drinking outcomes.

**Results:**

Overall, the results show that 16 out of the 31 pharmacological and psychotherapeutic alcohol treatment studies showed that being a non-smoker or decreased tobacco consumption during AUD treatment is associated with beneficial drinking outcomes, including reduced drinking, later relapse and prolonged alcohol abstinence. As such, smoking predicts poorer drinking outcomes in alcohol treatments. In the stop-smoking studies in patients with AUD, reduced smoking had virtually no effect on drinking behaviours. The inverse association between smoking and drinking outcome observed here indicates that non-smokers may be more successful to attain alcohol abstinence than smokers do. However, this association does not imply *per se* that smoking triggers alcohol consumption, since it can also mean that alcohol consumption promotes smoking.

**Conclusions:**

It is concluded that (continued) tobacco smoking may have a negative moderating effect on the treatment outcome of AUD treatments. To optimize treatment outcome of AUD one may consider informing and counselling patients with AUD about the risks of smoking for treatment outcomes and offering support for smoking cessation.

## INTRODUCTION

Excessive alcohol use and tobacco smoking are both major public health issues associated with considerable morbidity and mortality (Hurt *et al*., 1996). Moreover, smoking is associated with an increased risk of alcohol use disorders (AUD) (Hertling *et al*., 2005; McKee and Weinberger, 2013). For instance, smoking adolescents showed a 4.5-fold (95% CI: 3.1–6.6) higher vulnerability to develop AUDs than never-smokers who drank similar quantities (Grucza and Bierut, 2006). Conversely, heavy alcohol users or those who meet criteria for an AUD are more frequently current and former smokers (Lasser *et al*., 2000; McKee *et al*., 2007). Cross-sectional data from the US adult population showed that 39.2% and 64.9% of adults with a lifetime AUD diagnosis, and 51.0% and 64.5% of past-year AUD diagnosis were current and life-time smokers, respectively (Smith *et al*., 2014). Smoking patients with AUD drink more frequently and consume more alcohol on drinking occasions than non-smoking patients with AUD leading to a substantially greater lifetime exposure to alcohol (York and Hirsch, 1995). The smoking rate of individuals in treatment for AUD is considerably higher compared with the general population (70–95% and 23%, respectively) (Batel *et al*., 1995; Bobo *et al*., 1998; Kalman *et al*., 2005; Guydish *et al*., 2016). Moreover, the daily frequency of smoking is correlated to the amount of alcohol consumed and the severity of alcohol dependence (Batel *et al*., 1995). In the USA, current smoking is more than twice as prevalent in persons with AUD (38%) and heavy alcohol use (49%) than in persons without AUD or without heavy alcohol use (18% and 19%, respectively) (Weinberger *et al*., 2017a; Weinberger *et al*., 2019). Depending on geographic region, the prevalence of smoking ranges from 50% to at least 75% among individuals seeking treatment for AUD (Daeppen *et al*., 2000). Similarly, in Australia in 2007, 61% of patients with AUD smoked compared with 22% of persons in the general population (Kelly *et al*., 2012).

Analysis of data collected in the longitudinal NESARC (National Epidemiologic Survey on Alcohol and Related Conditions) study also showed that 3 years after achieving of abstinence from alcohol, past-year cigarette smoking was significantly associated with a diagnosis of AUD and recurrence of AUD symptoms (Dawson *et al*., 2007; Harrison and McKee, 2011). Later NESARC-studies showed that continued smoking constitutes an important risk factor for relapse in alcohol use. Subjects who had been treated for a substance-related disorder (SUD) but continued smoking had a significantly higher risk to relapse into drug and alcohol use (Weinberger *et al*., 2017b), whereas quitting from smoking significantly reduced the risk of AUD 3 years later (aOR = 0.7) (Cavazos-Rehg *et al*., 2014).

Green and Levy (1976) already said that ‘it is practically impossible to cure an alcoholic (or problem drinker) so long as he continues to smoke’. In former smoking subjects successfully treated for AUD, high urges to smoke cigarettes was associated with relapse to alcohol consumption (Cooney *et al*., 2007) and subjects with AUD had lower urges to drink and used less alcohol on days they did not smoke compared with days they smoked (Cooney *et al*., 2015).

Various evidence-based treatments for AUD are currently available, but regardless of the type of intervention, relapse rates in the first year following treatment are high, ranging from 60% to 90% (Kirshenbaum *et al*., 2009; Witkiewitz, 2011; Merkx *et al*., 2013; Chiappetta *et al*., 2014; Durazzo and Meyerhoff, 2017). Epidemiologic data have clearly shown a relationship between smoking status and AUD, but an overview on the (moderating) effect(s) of smoking on the outcome of AUD treatment is not available yet.

Two previous reviews (Apollonio *et al*., 2016; Thurgood *et al*., 2016) showed no consistent evidence that tobacco cessation interventions [e.g. nicotine replacement therapy (NRT)] have a positive effect on alcohol use outcomes. In the review by Thurgood *et al*. (2016), the impact of smoking cessation interventions in adult smokers recently or currently receiving for substance use disorders for 17 RCTs was reported. Substance use outcomes was reported in only 10 of the 17 RCTs: only two of these 10 RCTs showed some evidence for improved SUD outcomes, while the remaining eight RCTs showed no group difference in SUD outcomes. None of the RCTs suggested a negative effect of smoking cessation treatment on substance use outcomes. The review and meta-analysis of Apollonio *et al*. (2016) included 35 RCTs investigating the effect of smoking cessation interventions in subjects in treatment for or recovery from SUD. Their analysis showed that NRT was effective to stop smoking in both patients with AUD (RR = 1.47) and patients with other drug dependencies (RR = 1.85). In only 11 RCTs the effect of smoking cessation interventions on alcohol or drug use was studied. In these studies, NRT (vs. placebo) was not associated with higher abstinence rates from alcohol and other drugs (RR = 0.97; 95% CI: 0.91–1.03). Although these two reviews (Apollonio *et al*., 2016; Thurgood *et al*., 2016) suggest that smoking cessation interventions have no effect on the reduction in or the abstinence from alcohol or drug use, these reviews provide no information on the (moderating) effect(s) of (changes in) smoking behaviour on the outcome of AUD treatments. Therefore we performed a systematic review on studies investigating the effect of smoking status and smoking cessation on treatment success in patients with AUD. Since long-term effects are especially interesting in chronic relapsing illnesses like AUD, our review will focus on the effect of smoking and smoking cessation (treatments) on relapse to alcohol consumption during the follow-up of AUD treatment.

## METHODS

The current review only includes studies on persons with AUD with or without tobacco smoking, but no other SUDs. A person who abuses multiple drugs shows poorer treatment adherence and may encounter more difficulties in stopping drinking and has a higher risk for relapse to alcohol use after treatment (Miller and Bennett, 1996; Mason and Lehert, 2009). Persons with AUD and comorbid other psychiatric disorders were also excluded because they are significantly less successful in smoking cessation (Ziedonis *et al*., 2008), e.g. depressive episodes predicted drinking relapse among subjects with AUD (Hasin *et al*., 2002).

Using the PRISMA-protocol, a systematic search was performed on 25 January 2022, to retrieve eligible studies in PubMed about the relation of smoking status and smoking cessation with treatment success in AUD studies since 1990. The most recent publications i.e. those ‘in process’ were retrieved by adding the term ‘NOT medline’ to the search string. In addition, using the combination of AUD and smoking, six completed studies with reported results were retrieved from ‘Clinicaltrials.gov’.

Inclusion criteria for eligible studies were that (a) subjects included in the study were patients diagnosed with AUD (i.e. not heavy-drinking subjects or problematic drinkers who were not diagnosed with AUD) and (b) information was available on smoking status. Exclusion criteria were: (a) studies written in a language other than English, German, French and Dutch, (b) studies performed in patients with AUD with co-morbid other psychiatric disorders, (c) inclusion of subjects with a combined alcohol and illicit drug use disorder or subjects using substances other than alcohol and nicotine (cf. search string in Appendix) and (d) studies with a sample size <40 patients. Relapse is defined as resumed drinking.

**Fig. 1:**
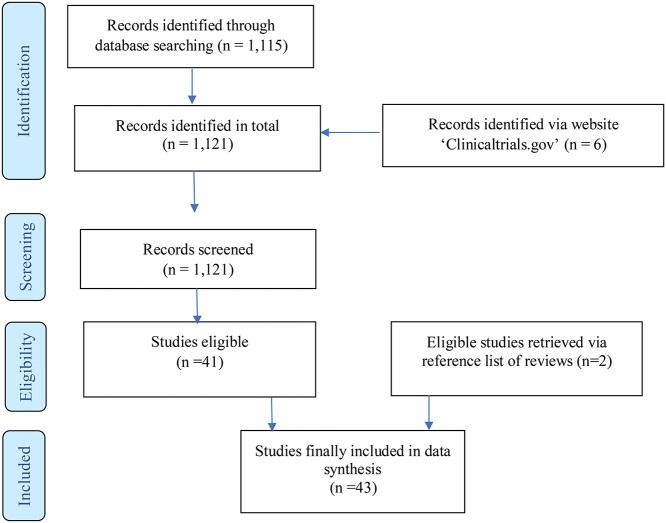
PRISMA flow diagram.

The selection of appropriate studies was performed by JvA and WvdB in two rounds. A total of 1115 publications were identified from the initial search, including six studies from ‘Clinicaltrials.gov’. The title and abstract of these studies were screened to determine eligibility, which resulted in 43 eligible studies. Using the reference lists of published meta-analyses and reviews, two additional eligible studies were identified and included. The final sample included therefore 43 studies. Figure 1 shows the PRISMA flow chart for the identification, screening and inclusion of the studies. See Supplement for search string and PRISMA checklist.

## RESULTS

Tables 1–3 summarize the main data of the 43 studies, covering 10,296 patients, included in this systematic review. The studies are categorized in studies applying treatment of AUD with either cognitive behavioural programmes (Table 1) or pharmacotherapy (Table 2), and studies on the effect of NRT for nicotine dependence in patients with AUD on drinking outcomes (Table 3). Outcome was mainly focussed on relapse and drinking behaviour at follow-up in association with smoking status or changed smoking behaviour.

**Table 1:** Effect of smoking on drinking outcomes during treatment of AUD using variants of psychotherapy (PT) or at follow-up (FU). Relapse is defined as resumed drinking

N	Type	Subjects	Outcome at follow-up (FU), specifically with respect to effects of smoking on drinking	Association^#^	Reference
1742	Longitudinal survey	1091 SM*, 651 NSM	At 1-year. FU: abstinence in smokers was lower than in non-smokers (aOR = 0.60; 95% CI: 0.43–0.83). Effect size: 1/0.6 = 1.6	Positive 1.6	(Ahmedani *et al*., 2012)
94	Longitudinal survey	85 SM, 9 NSM	Relapse at 5-year. FU: of smokers 28.1%, quit smoking: 7.5%; non-smokers: 10%. Effect size: 28.1/7.5 = 2.8	Positive 2.8	(Sobell and Sobell, 1996)
129	Cohort study+FU	57 SM, 65 NSM	Relapse at 18-month. FU: SM: 87%; NSM: 52%, *P* = 0.003. Effect size: 87/52 = 1.7	Positive 1.7	(Durazzo and Meyerhoff, 2017)
955	RCT	All SM	Project MATCH (three different PT). At 15-month. FU: less relapse in subjects who reduced smoking than those who smoked more or unchanged (Wilcoxon χ^2^ = 7.5, *P* = 0.02). Association between no drinking and smoking at 1 year. FU: decreased smoking: 28%; unchanged smoking: 21% and increased smoking: 20% (OR = 1.3). Effect size: 1.3	Positive 1.3	(Friend and Pagano, 2005a)
160	RCT	All quitted smoking	Project MATCH (three different PT). Quitters (subsample of previous study) reported an increase of 8% days abstinence (84% days vs. 79% days; *P* < 0.05). Effect size: 84/79 = 1.1	Positive 1.1	(Friend and Pagano, 2005b)
124	Cohort study+FU	moderate -heavy SM: 79; NSM: 46	Being a non-smoker at treatment entry predicted alcohol abstinence 7 years later (56.1% vs. 25.5%; *P* = 0.001). Effect size: 56.1/25.5 = 2.2	Positive 2.2	(Hintz and Mann, 2007)
279	RCT	224 SM, 75 NSM	Smoking increased the risk for alcohol relapse (HR: 4.0; 95% CI: 1.58–9.92, *P* = 0.0033). Effect size: 4.0	Positive 4.0	(Hufnagel *et al*., 2017)
144	Cohort study+FU	144 SM	At 6 month. FU: abstinence rate of quitters of smoking and continuing smokers was 93% and 62%, respectively (χ*^2^* = 5.19, *P* < 0.05). Effect size: 93/62 = 1.5	Positive 1.5	(Karam-Hage *et al*., 2005)
95	Cohort study+FU	42 SM, 32 former SM, 21 NSM	At 6-month. FU: active smokers were 2.6 times more likely to relapse than non-smokers (OR = 0.39 (95% CI: 0.01–1.56, *P* = 0.18) and 6.6 times more likely than former smokers (OR = 0.15; 95% CI: 0.04–0.52, *P* = 0.003). Effect size: 1/0.15 = 6.7	Positive 6.7	(Nguyen *et al*., 2020)
598	RCT	315 SM, 283 NSM	At 5-year. FU: abstinence from alcohol in the prior 30 days was 48.3% for SM and 64.0% for NSM (χ*^2^* = 14.9, *P* < 0.001. Effect size: 64.0/48.3 = 1.3	Positive 1.3	(Satre *et al*., 2007)
61	RCT	Lapsers analysed: 32 (smoking) and 29 (drinking)	Smokers were 6 times more likely to have a drinking lapse between 2 weeks before quitting and 2 weeks thereafter than those not smoking (Wald χ2 = 15.03, df = 1, *P* < 0.001, OR = 6.0, 95% CI: 2.44–15.09, *P* < 0.001). Effect size: 6.0	Positive 6.0	(Holt *et al*., 2012)
155	Cohort study+FU	155 SM	At 1-year. FU: changed smoking pattern did not result in differences on the drinking measures (i.e. abstinent days, low drinking days, and heavy drinking days)	No	(Toneatto *et al*., 1995)
347	Cohort study+FU	128 SM, 63 NSM, 47 former SM, 109 regular snuffers	At 2.5-year. FU: type of smokers regular snuffers, regular smokers, NSM and former tobacco users) showed no differences in total abstinence or days of alcohol use per week in the last 30 days	No	(Rauwolf *et al*., 2017)
499	RCT	381 white SM, 78 African American SM	Treatment with PT for smoking either concurrently (CON) or delayed (DEL; 6 months after Minnesota for AUD). CON vs. DEL: at 18-months. FU, no difference in smoking cessation and at 12-months. FU: alcohol abstinence rate among white Americans: CON (43%) lower compared with DEL (50%); *P* = 0.05), but no differences among African Americans	No	(Fu *et al*., 2008)
116	Cohort study+FU	116 SM	At 6-months. FU, the change in smoking rate did not significantly interact with relapse status	No	(Gulliver *et al*., 2000)
138	RCT	47 SM, 91 NSM (all females)	At end-of-treatment: SM showed greater reduction in drinks per drinking day compared with NSM (B = 1.6, SE = 0.78, t(60.87) = 2.10, *P* = 0.038). Effect size: - 1.6	Negative −1.6	(Bold *et al*., 2020)

* SM: smoking; NSM: non-smokers; ^#^ Association between smoking and alcohol use; figure indicates the effect size. OR: odds ratio; RCT: randomized controlled trial.

**Table 2:** Effect of smoking on drinking outcomes during pharmacological treatment of AUD or at follow-up (FU). Relapse is defined as resumed drinking

N	Type	Drug^1^	Outcome at follow-up (FU), specifically with respect to effects of smoking on drinking	Association^#^	Reference
*Harm reduction (reduced alcohol consumption)*					
249	Cohort study + FU	Bupropion: 249	At 12-wk. FU, any smoking was more prevalent on a heavy drinking day (38.5%) than on a non-heavy drinking day (19.1%; *t* (69) = 4.57, *P* < 0.001). Effect size: 38.5/19.1 = 2.0	Positive 2.0	(Leeman *et al*., 2008)
40	RCT 12 weeks	Varenicline: 19; placebo: 21	In the course of treatment with varenicline, SM reduced smoking by 37% (χ*^2^* = 4.52, *P* = 0.03) and had less heavy drinking days (beta = −2.3, χ*^2^* = 6.20, *P* = 0.01). Effect size: 2.3	Positive 2.3	(Plebani *et al*., 2013)
131	RCT 16 weeks	Varenicline: 64; placebo: 67	Varenicline increased smoking abstinence in weeks 13–16: varenicline 13% vs. placebo 0% (*P* = 0.003). Varenicline reduced drinking in man: no heavy drinking days in weeks 9–16: varenicline 29% vs. placebo 6% (Cohen h = 0.64; 95%CI: 0.22–1.03), but no effect in woman (Cohen h = −0.60; 95%CI: −1.21 to 0.04). Effect size (men): 29/6 = 4.8	Positive in men (4.8); negative in women	(O'Malley *et al*., 2018)
196	RCT 13 weeks	Varenicline: 95; placebo: 101	At 13-weeks FU: compared with placebo, varenicline + reduced smoking rate reduced percent heavy drinking days (PHDD) (55.4 vs. 36.1, respectively; *P* = 0.04), whereas varenicline + same or increased smoking had no effect on PHDD (51.6 vs. 68.3, respectively; *P* = 0.15). Effect size: 55.4/36.1 = 1.5	Positive 1.5	(Falk *et al*., 2015)
131	RCT 16 weeks FU: 6, 9, 12	Varenicline: 64; placebo: 67	At 4, 9 and 12-month. FU: males had higher rates of no heavy drinking days with varenicline (28.8%) vs. placebo (6.4%, *P* = 0.004) and females, but not males, showed higher rates of abstinence for varenicline (varenicline: 21.0% vs. placebo, 0.0%, *P* = 0.05)	Mixed positive findings	(Bold *et al*., 2019)
146	RCT 16 weeks; 12 and 16 weeks FU	NAL: 73; placebo: 73	At 16-wk. FU, significant difference between NAL (12.0%) and placebo (36.5%) in percent heavy drinking days (PHDD) (F = 6.40, df 1,41 *P* = 0.02), but this was not due to less daily smoking because there was no difference in smoking rate between NAL and placebo (F = 1.35, df 1,38 *P* = 0.25)	No	(Anton *et al*., 2018)
196	RCT 13 weeks	Varenicline: 95; placebo: 101	Varenicline reduced % heavy drinking days compared with placebo (37.9 vs. 48.4, respectively; *P* = 0.03), but no interaction treatment x smoking status at baseline (*P* = 0.96)	No	(Litten *et al*., 2013)
128	RCT and 3-month. FU	Mecamylamine: 65; placebo: 63	At 3-month. FU: for drinks per drinking day and % of (heavy) drinking days and % no difference between (a) mecamylamine (a nicotinic acetylcholine receptor antagonist) vs. placebo or (b) between SM vs. NSM (*P* = 0.14, *P* = 0.29 and *P* = 0.43, respectively)	No	(Petrakis *et al*., 2018)
616	RCT 16 weeks	NAL: 309; placebo: 307	NAL was more effective to reduce (a) days abstinent from drinking in SM (mean = 78.4 ± 1.0) than in NSM (mean = 74.0 ± 2.0; *P* = 0.004) and (b) number of drinks per drinking day in SM (mean = 13.6 ± 1.0) than in NSM (mean = 9.7 ± 0.9; *P* = 0.01). Effect size SM vs. NSM: 78.4/74.0 = −1.1 and 13.6/9.7 = −1.4, respectively	Negative - 0.94 to −0.71 (NAL: 1.1 to 1.4 fold more effective in SM vs. NSM)	(Fucito *et al*., 2012)
44	Pre-post 12 weeks	Placebo: 44	At 6-month. FU: alcohol consumption (drinks/day) was lower in SM (0.20) than in NSM (0.67; *P* = 0*.*012), and abstinence-rates were 72% for SM and 38% NSM. Time to first relapse to drinking was longer in SM than in NSM: hazard ratio = 2*.*26; *P* = 0*.*036. Effect size: 1/2.26 = −0.4	Negative - 0.4	(Schmidt and Smolka, 2007)
*Relapse to alcohol drinking (abstinence rate)*					
155	RCT 12 weeks	NAL: 49; topiramate: 52; placebo: 54	NSM extended first relapse compared with SM (7.09 ± 4.98 vs. 5.42 ± 4.84 weeks; (*t* = 2.02, 153 df, *P* = 0.04). Corrected for type of medication, smoking increased relapse to drinking by 65% (Wald = 4.93, 1 df, *P* = 0.03, OR = 1.7; 95%CI: 1.06–2.56). Effect size: 1.7	Positive 1.7	(Baltieri *et al*., 2009)
220	RCT 6, 12, and 18 months FU	NAL: 220;	213 provided relapse data for first 13 weeks At 3-month. FU, NAL significantly (*P* = 0.05) predicted rate and time of drinking relapse; relapse in placebo was 2-fold vs. NAL, but smoking had no significant effect on relapse to heavy drinking in the overall sample	No	(Gelernter *et al*., 2007)
63	RCT and 12-months FU	Lisuride: 63; placebo: 57	At 12-months FU: no difference in alcohol abstinence rate between 48 SM and 15 NSM in lisuride group (33% vs. 20%). However, SM tended to be longer abstinent than NSM (173 vs. 114 days; *P* = 0.092)	No	(Schmidt and Smolka, 2001)
94	RCT 12 weeks And 12-weeks FU	Topamirate: 45; placebo: 49	At 12-weeks FU: topiramate: more smoking associated with lower alcohol abstinence (% of days abstinent) (*r* = − 0.24; *P* = 0.01); placebo: more smoking was associated with higher alcohol abstinence (*r* = +0.28; *P* = 0.003)	Mixed result	(Johnson *et al*., 2005)
557	Pre-post 24 weeks	Acamprosate 557	At 6-months FU; abstinence rates were 38% for SM vs. 28% for NSM (*P* < 0.015). Time to first relapse to drinking was longer in SM than NSM: hazard ratio = 1.34; *P* = 0.015. Effect size: 1/1.34 = −0.7	Negative - 0.7	(Schmidt and Smolka, 2007, study II)

^1^NAL: naltrexone. ^#^ Association between smoking and alcohol use; figure indicates the effect size. * SM: smoking; NSM: non-smokers; NAL: naltrexone.

**Table 3:** Effect of smoking, following NRT, CBT, bupropion or combinations thereof, on drinking outcomes during treatment or at follow-up (FU) in randomized controlled trials (RCT) in subjects with AUD. Relapse is defined as resumed drinking

N	Subjects^1^	Main results, specifically with respect to effects of reduced smoking rate on drinking outcomes	Association^#^	Reference
*Harm reduction (reduced alcohol consumption)*				
90	Tx: 60; CTR: 30	Tx: Minnesota. At 6-months FU, (a) smoking rate and quit attempt rates in Tx and CTR was nearly identical (97% and 94%, respectively), (b) drinking since treatment discharge was higher in CTR (35%) than in Tx (17%; *P* > 0.05) and (c) significant reduction in relapse to drinking in Tx (aOR = 0.15; 95% CI: 0.02–0.89), implying no association between reduced smoking and reduced drinking	No	(Bobo *et al*., 1996)
444	Tx: 218; TAU 226	Tx: individual counselling. At 12-months FU, Tx had no effect on quitting rate (54% vs. 49%), but reduced both moderate drinking (24.3 vs. 34.1%) and heavy drinking (33.0 vs. 37.1%) and increased abstinence 42.7 vs. 28.8%; OR = 1.84, 95%CI: 1.28–2.92), implying no association between reduced smoking and reduced drinking	No	(Bobo *et al*., 1998)
102	Tx: 102	Tx: concurrent alcohol and tobacco treatment (CBT + NRT) vs. brief counselling. Evaluation using 14 days of electronic diary (ED) assessments after discharge from treatment: smoking status (smoking, not abstinent) was not associated with ED-ratings of positive drinking urges (−0.75, *P* > 0.40), but positive urge to drink was higher after smoking a cigarette than before smoking	No	(Cooney *et al*., 2007)
96	Tx, 1: 45; Tx, 2: 51	Tx: NRT At 1-year FU, NRT gave better smoking outcomes than placebo, but alcohol outcomes (alcohol abstinence, time to first drink and time to first heavy drinking day) were not significantly different across medication conditions	No	(Cooney *et al*., 2009)
151	CSC: 105; DSC: 46	Tx: concurrent smoking cessation (CSC) or delayed smoking cessation (DSC). At 13 weeks After starting treatment, smoking abstinence was 19.0% (CSC) and 0% (DSC). No difference for self-reported proportion days heavy drinking. However, on non-smoking days subjects in the CSC-group consumed lower numbers of drinks and had lower urge to drink	No	(Cooney *et al*., 2015)
115	Tx: not specified	Tx: NRT. At 6-months FU, NRT was still effective (24% vs. 6%; OR = 4.9; *P* = 0.02). None of the study participants reported drinking problems or increases in craving for alcohol	No	(Hughes *et al*., 2003)
103	Tx: 53; CTR: 50	Tx: CBT; CTR (autogenic training). Only 44 patients were available for 6-months FU smoking and alcohol outcomes. No difference between groups for either smoking cessation or self-reported alcohol abstinence and alcohol use in the past 7 days	No	(Mueller *et al*., 2012)
58	Tx: 30; Ref: 28	Tx: bupropion + NRT; Ref: NRT. At 6 months: discontinued smokers reported greater continuous abstinence from alcohol, fewer drinks per day, and more abstinent days in the previous 30 days but, the differences were not statistically significant	No	(Grant *et al*., 2007)
*Relapse to alcohol drinking (abstinence rate)*				
130	Tx: 130	Tx: NRT. Nicotine abstinence at 24 weeks FU was related to a longer length of alcohol abstinence (OR = 1.6, *P* = 0.003)	+ 1.6	(Kalman *et al*., 2006)
162	Tx: 82; TAU: 80	Tx: CBT + NRT; TAU: smoking cessation counselling. At 26-weeks FU: CBT + NRT had higher smoking quit rate than TAU (*P* = 0.03), but the difference was not significantly different at 38 or 52 weeks No differences in alcohol abstinence between the two groups at any FU	No	(Carmody *et al*., 2012)
83	Tx: 42; Ref: 41	Tx: CM (CBT + NRT + CM); Ref (CBT + NRT). Confirmed quit rate: CM: 60% vs. Ref: 29%. At 6-months FU: smoking abstinence was not related to combined alcohol and drug abstinence (*b* = 0.01, 95%CI: −0.17-0.27, *P* > 0.05)	No	(Cooney *et al*., 2017)
110	Tx: 56; placebo: 54	Tx: bupropion. At end-of-treatment (wk. 52): no difference between the groups for smoking abstinence, 41.1% (95% CI: 28.1%–55.0%) vs. 40.7% (95% CI: 27.6%–55.0%), respectively. 4% of subjects (*n* = 4) relapsed to alcohol	No	(Hays *et al*., 2009)
205	Tx1: 72; Tx2: 63; TAU: 70	Tx1: CBT; Tx2: CBT + NRT. Recovering subjects with an alcohol disorder who were >3 months abstinent from alcohol and drugs. At 12-months FU: no difference in quit rate (all 27%). Only 4% (7 of 188) relapsed to alcohol or drugs. Alcohol relapse did not differ by treatment group or smoking status	No	(Martin *et al*., 1997)

^1^Tx: treatment group; TAU: treatment as usual; CTR: control group; ^#^ Association between smoking and alcohol use; figure indicates the effect size; aOR; adjusted odds ratio; CM: contingency management.

Table 1 covers 15 studies including 5542 treated for AUD with cognitive behavioural or similar interventions showing that—except for four studies showing no effect of smoking on drinking outcomes and one study (Bold *et al*., 2020) with a negative association—(more) smoking or not quitting from smoking was associated with worse drinking outcomes in 10 studies. Compared with non-smokers, the risk of smokers to drinking relapse within 6 months follow-up was generally 1.3 to 2.8 times higher with two studies showing an even higher risk of 3.96 (Hufnagel *et al*., 2017) and 6.0 (Holt *et al*., 2012).There was one exception to this general pattern of a positive or no association: female smokers showed larger reductions in drinking behaviour than their non-smoking peers (mean change of 4.0 ± 5.6 drinks vs. 2.4 ± 3.4 drinks; *P* = 0.038) (Bold *et al*., 2020). It should be noted, however, that the smoking females had greater baseline alcohol severity (more DSM-IV AUD symptoms) than their non-smoking peers and no statistical adjustment was performed and, thus, selection bias and confounding cannot be excluded (Bold *et al*., 2020).

Table 2 covers 15 studies including 2966 patients treated for AUD with pharmacotherapy. Smoking was positively associated with a 1.5 to 2.3 higher risk of drinking relapse in six studies, while in six studies no significant difference between smokers and non-smokers was observed with respect to alcohol abstinence and drinking behaviour. In one study the results were mixed dependent on the treatment condition and in three studies there was a negative association with smokers having more successful alcohol outcomes than non-smokers. In two of these studies with a negative association, time to first relapse was longer in smokers than in non-smokers (Schmidt and Smolka, 2001; Schmidt and Smolka, 2007) and in another study (Fucito *et al*., 2012) naltrexone was more effective to reduce drinking in smokers than in non-smokers. Varenicline was used in five treatment studies of AUD, of which three studies were positive (Plebani *et al*., 2013; Falk *et al*., 2015; O'Malley *et al*., 2018) i.e. a concomitant reduction of both smoking as well as drinking was observed.

Table 3 covers 13 studies where 1849 smoking patients with AUD had been treated with NRT, cognitive behavioural therapy (CBT), bupropion or combinations thereof to reduce or quit smoking. In seven studies the treatment successfully reduced smoking at follow-up, while the remaining six studies were negative in this respect. Only in the study of Kalman *et al*. (2006), the intervention reduced both smoking and drinking, implying a significant association between both (OR = 1.6). In two studies, drinking was reduced in the intervention group i.e. those treated to reduce or quit smoking, compared with placebo with ORs values of 1.2 and 1.8, but the intervention failed to significantly reduce smoking (Bobo *et al*., 1996; Bobo *et al*., 1998), implying no association between smoking and drinking. Of the remaining 10 smoking cessation studies: (a) six studies resulted in reduced smoking at follow-up, but this was not associated with reduced drinking and (b) four studies showed no effective reduction in smoking. Finally note that the studies focusing on harm reduction (reduced alcohol consumption) as outcome generally showed similar results to those focusing on relapse to alcohol drinking (cf. Tables 2 and 3).

In summary, the outcome of 16 of the 30 studies regarding the effect of smoking on the success of behavioural or pharmacological treatment of AUD showed that smoking cessation or decreased smoking frequency was associated with lower relapse rates to alcohol or more drinking at follow-up, whereas in only four studies the effect was in the opposite direction. Only one of the 13 NRT studies showed a positive association between smoking abstinence and alcohol abstinence.

## DISCUSSION

The current review shows that in about half of the 30 treatment studies being a non-smoker or decreased tobacco consumption during AUD treatment is associated with beneficial drinking outcomes, including reduced drinking and alcohol abstinence (cf. Tables 1 and 2). As such, smoking predicts poorer treatment outcome in quitting alcohol, considering that it was associated with a higher relapse probability, or early relapse after treatment in individuals with AUD recovering from drinking. Four of the 31 studies showed the reverse association i.e. smoking was negatively associated with drinking relapse. For instance, in the study of Bold *et al*. (2020), female smoking AUDs showed greater reductions in alcohol consumption, which may be due to their more severe baseline AUD symptoms compared with their non-smoking peers. In another study, naltrexone was more effective to reduce drinking in smokers vs. non-smokers (Fucito *et al*., 2012). However, in this study, smokers were more often male, more severely alcohol dependent and reported more drinking consequences and a higher percentage of abstinent days prior to commencing treatment than non-smokers, which may explain the positive association between smoking and treatment success. The results of two other pharmacotherapy trials (Schmidt and Smolka, 2001; Schmidt and Smolka, 2007) also showed a negative association in that smokers were longer abstinent from alcohol than non-smokers. However, the smoking effect for preventing relapse was modest, and—like in the Fucito *et al*. (2012) study—smokers at base-line were more often male, had begun heavy drinking earlier and had consumed about twice as much alcohol drinks at index time compared with their non-smoking peers.

With respect to pharmacotherapeutic treatment of AUD the results obtained with varenicline are of specific interest. A recent network meta-analysis showed that varenicline plus NRT was most effective for sustained smoking abstinence (OR = 5.8; 95% CI: 2.3–14.9) (Thomas *et al*., 2021), while varenicline also significantly reduced drinking (% heavy-drinking days, drinks per day and alcohol craving) compared with placebo (Litten *et al*., 2013). As such, varenicline may serve as a promising option for AUD treatment of smoking individuals. Indeed, varenicline induced a concomitant reduction of both smoking and drinking in three out of five AUD treatment studies (Plebani *et al*., 2013; Falk *et al*., 2015; O'Malley *et al*., 2018), endorsing the negative impact of smoking on treatment outcome in AUD.

Collectively, the current findings confirm the previous finding that a positive treatment outcome in AUD is facilitated by quitting smoking and countered by relapse to smoking (Mason and Lehert, 2009; McKee *et al*., 2009; Weinberger *et al*., 2013). In addition, the current review endorses the observation that cigarette smoking (daily and non-daily) was positively associated with alcohol abuse and dependence 3 years later compared with non-smoking (Weinberger *et al*., 2015).

However, the observed association between smoking and drinking outcome does not imply that smoking is a trigger for alcohol consumption, since it can also mean that alcohol consumption promotes smoking. Two following observations may be relevant here: (a) in subjects with a current or past AUD, the probability of any smoking was significantly higher on a heavy drinking day (38.5%) than on a non-heavy drinking day (19.1%, *P* < 0.001) (Leeman *et al*., 2008), (b) among smoking adolescents alcohol drinking was strongly associated with smoking lapses (any use after achieving 24-h smoking abstinence), but not with a relapse of smoking (Van Zundert *et al*., 2012) and (c) in treatment seeking patients with AUD, the urge to drink was positively associated with the urge to smoke (Gulliver *et al*., 2000). That is, urge to drink may be a conditioned stimulus for the urge to smoke. The results of these studies (a–c) indicate that alcohol use may trigger smoking. This is supported by our observations regarding NRT treatment of smoking patients with AUD (cf. Table 3) showing that smoking cessation or reduced smoking was not consistently associated with reduced alcohol consumption or alcohol abstinence. This contradicts the observations that (a) high urges to smoke cigarettes in former smoking subjects successfully treated for AUD are associated with relapse to alcohol consumption (Cooney *et al*., 2007), and (b) subjects with AUD have lower urges to drink and use less alcohol on days they do not smoke compared with days they smoke (Cooney *et al*., 2015). However, Cooney *et al*. (2003) previously demonstrated in alcohol-dependent smokers that (a) while non-deprived, alcohol cue presentations were associated with significant increases in urges to drink and urges to smoke, and (b) acute nicotine deprivation increased smoking urges, but not urges to drink, indicating that smoking cessation is unlikely to increase the risk of relapse to alcohol in alcohol dependent patients (Cooney *et al*., 2003).

With respect to treatment of smoking in subjects with AUD, a recent meta-analysis (nine RCTs, 908 smokers with AUD) (Guo *et al*., 2021) showed that varenicline significantly reduced short-term smoking (three RCTs, OR = 6.27; 95% CI: 2.49–15.78; *P* < 0.05), while naltrexone, bupropion and topiramate had no significant effect on short-term smoking cessation. The study did not report on potential effects of smoking cessation interventions on drinking outcomes in this patient population. However, two previous reviews did focus on the effect of smoking cessation interventions on alcohol and drug consumption in patients treated or in recovery from SUD (Apollonio *et al*., 2016: 35 RCTs; Thurgood *et al*., 2016: 17 RCTs). Their results suggested that tobacco cessation interventions (e.g. NRT) in patients treated for AUD or SUD have no effect on alcohol or other drug use outcomes. However, only few of the included RCTs were on AUD and alcohol use outcomes and more importantly, these reviews contained no information on the (moderating) effect(s) of (changes in) smoking behaviour on the outcome of AUD treatments.

The results of the current review, obtained in a more specific group of patients with AUD and with another focus, indicate that smoking has a negative effect on alcohol outcomes in patients treated for AUD, but (similar to the reviews of Apollonio *et al*., 2016 and Thurgood *et al*., 2016) that treatment of smoking in patients with AUD—resulting in reduced smoking or quitting smoking—does not consistently lead to better alcohol outcomes.

There are several explanations for the high sustained smoking rate among patients with AUD (Lien *et al*., 2021). Nicotine dependence and AUD may have a shared genetic predisposition (Hancock *et al*., 2018) resulting in both nicotine and alcohol triggering the dopamine release in the mesolimbic dopamine pathway, which mediates the rewarding and reinforcing properties of both drugs. Furthermore, patients with AUD often suffer from mental health problems, and tobacco smoking is often used as a form of self-medication to relieve psychiatric symptoms (Lien, 2016). In addition, smoking counteracts the sedative and cognitive effects of alcohol and softens the withdrawal symptoms of alcohol (Mendelsohn and Wodak, 2016).

At present, several proven-effective cognitive behavioural interventions and pharmacotherapies to effectively treat AUD are available (Akbar *et al*., 2018; Farokhnia *et al*., 2019), but smoking continues to negatively affect the success of AUD treatment. Since the introduction of varenicline, topamirate and bupropion around 2005, no new promising drugs have been developed to treat AUD. Perhaps, psychedelics provide new opportunities for smoking subjects with AUD, considering that psilocybin may be effective against alcohol dependence as well as against smoking (van den Brink *et al*., 2020).

Current findings contribute to a better understanding of the treatment of AUD in which specific factors are associated with relapse and those that prevent relapse. Such factors may have a role in a personalized medicine framework to improve patient outcomes.

### Strengths and limitations

A major methodological strength of this systematic review is the selection of subjects which were limited to AUD only, although this may also be seen as a limitation since many patients in real practice are polydrug users and psychiatric comorbidity is the rule rather than the exception. Another limitation is that AUD relapse is known to be associated with a large variety of moderators, including AUD severity, age, gender, inpatient/outpatient, type of cognitive behavioural treatment, type of pharmacotherapy, variation in follow-up and certain social factors (Sliedrecht *et al*., 2019) and for many of these factors no adjustments were performed in the eligible studies of this review. Furthermore, in the 43 included studies, men are in the majority (71.6% of total sample). The small number of female patients is a limitation and our results cannot be generalized to this important group of patients with AUD. Another important limitation of the current review is that we presented data from studies with very different designs and sample sizes and with different ways to estimate the influence of smoking on the alcohol outcomes of AUD treatments. For a proper causal interpretation of the influence of smoking on the alcohol outcomes of AUD treatments, RCTs with a pre-stratification for smoking status is needed. Unfortunately, no such studies were available for this review. Moreover, the studies on the effect of smoking status on drinking outcome were all based on group mean values, while monitoring the effect per individual (at individual level) would be more valuable and appropriate, considering the inter-individual variation within the samples. On the other hand, it appears that different studies with different designs, different AUD interventions and different alcohol outcomes came to the same conclusion: smoking has in general a negative influence on the alcohol outcomes of AUD treatment. Due to large differences across the studies in terms of design, sample composition and selection, and outcome measures, no meta-analysis and quality ratings of the studies are performed. For instance, with respect to smoking cessation, older age, female, having higher education, smoking rate and one’s own will to quit smoking have been identified as significant determinants of successful cessation (Ockene *et al*., 2000; Lee and Kahende, 2007; Marti, 2010; Kim, 2014; Smith *et al*., 2016).

## Funding

The authors received no financial support for the research, authorship and/or publication of this article.

## Declaration of conflicting interests

J.v.A. has no potential conflicts of interest with respect to the research, authorship and/or publication of this article. W.v.d.B. has a potential conflict of interest as a consultant for Lundbeck, D&A Pharma and Kinnov Therapeutics.

## Supplementary Material

Supplement_agac027
